# Influence of different endodontic treatment protocols on tooth survival: A retrospective cohort study with multistate analysis and group balancing

**DOI:** 10.1111/iej.14271

**Published:** 2025-06-13

**Authors:** Ahmed Elmaasarawi, Mohamed Mekhemar, Andreas Bartols

**Affiliations:** ^1^ Clinic for Conservative Dentistry and Periodontology Christian‐Albrechts‐University Kiel Kiel Germany; ^2^ Department of Restorative Dentistry October University for Modern Sciences and Arts (MSA) Cairo Egypt; ^3^ Dental Academy for Continuing Professional Development Karlsruhe Karlsruhe Germany

**Keywords:** group balancing, multistate analysis, root canal preparation [E06.397.778.889], root canal treatment, survival analysis [E05.318.740.998], treatment outcome [N04.761.559.590.800]

## Abstract

**Aim:**

This study aimed to assess how three different treatment protocols affect the survival of teeth and the survival of teeth without further interventions after root canal treatment (RCT), while also considering additional factors that could potentially influence the treatment outcome.

**Methodology:**

Data were collected from an outpatient clinic database from July 1999 to January 2024 and 14 233 treated teeth could be included in a retrospective cohort study. Treatment protocols incorporated hand files (Protocol 1), multiple‐file rotary NiTi systems added with passive ultrasonic irrigation (PUI), citric acid and occasionally chlorhexidine (Protocol 2) and reciprocating instruments added with PUI, EDTA, warm vertical compaction and calcium silicate‐based sealer (Protocol 3). Survival analysis coupled with Cox proportional hazard regression and Kaplan–Meier curves took into account several variables including treatment, patient demographics and experience of the treatment provider. Confounding was addressed by entropy balancing and gradient boosted logistic regression. Multistate analysis was conducted to evaluate the influence of treatment protocols on the transition between various intervention states.

**Results:**

Survival analysis revealed that Protocol 3 significantly enhanced survival rates and survival rates without further interventions by 30%–40% compared to both other protocols. Additionally, higher patient age was identified as a negative predictor of treatment outcomes. Supportive periodontal treatments were a positive predictor. Factors such as tooth type, vitality, number of visits, experienced treatment provider, calcium silicate‐based sealer and patient gender did not significantly affect outcomes in the adjusted models. Multistate analysis confirmed that Protocol 3 was associated with significantly reduced incidences of retreatment and extraction.

**Conclusion:**

Protocol 3 significantly enhanced survival and survival without further interventions compared to both other protocols. Patient age and supportive periodontal treatments were significant predictors of outcomes throughout all calculated models.

## INTRODUCTION

In recent years, significant advancements have been made in root canal treatment (RCT) protocols. While these improvements should have the potential to enhance the success and outcome of RCT, scientific proof on this matter is currently sparse (Gulabivala & Ng, 2023; Ng, Mann, & Gulabivala, 2008). Despite the relatively large number of studies on the outcome of endodontic treatment, few of these studies have investigated the effect of treatment protocols or different preparation instruments on the outcome (Ng, Mann, & Gulabivala, 2008, 2010). Moreover, most of these studies found no systematic evidence that the use of more recent treatment protocols improves endodontic treatment outcome (Fernández et al., 2017; Fleming et al., 2010; Mareschi et al., 2020). Since the development of rotary Nickel‐Titanium (NiTi) files, there have been only a few studies demonstrating an improvement in periapical healing as an indication of the success of root canal treatment (Bürklein & Arias, 2023; Cheung & Liu, 2009; Hoskinson et al., 2002; Mareschi et al., 2020). However, treatment protocols including reciprocating systems have demonstrated higher success rates in comparison with hand files in terms of survival without further interventions, while at the same time rotary instruments showed no significant differences to a treatment protocol with hand instruments but also not to a treatment protocol involving Reciproc instruments (Bartols et al., 2020). On the other hand, two recent randomized controlled trials concluded that the healing rates and discomfort associated with two different protocols incorporating manual and reciprocating instruments were not different after one year of follow‐up (de Figueiredo et al., 2020; Diniz‐de‐Figueiredo et al., 2020). Another study concluded that protocols utilizing rotary BioRaCe instruments compared to Reciproc instruments showed no difference in the success rates after one year (Neves et al., 2020).

Advancements and improvements in RCT protocols cover not just the preparation of the root canal but also essential steps like irrigation, obturation and disinfection techniques. While the shift from hand files to rotary NiTi systems and reciprocating systems has garnered significant attention, the progress made in these other steps might be just as vital for influencing treatment outcomes. For instance, irrigation protocols have evolved significantly with the introduction of passive ultrasonic irrigation (PUI), which was found to have a favourable effect on the outcome of RCT (Gobbo et al., 2024). In addition, it was suggested that the use of EDTA as an adjunct to sodium hypochlorite improves periapical healing by 1.3–2.3 odds (Ng et al., 2011a, 2011b). On the other hand, it seems that vertical condensation techniques have not improved the outcome of RCT when compared to the lateral compaction technique (Pirani & Camilleri, 2023). Moreover, a recent study demonstrated that there is no significant difference in treatment outcomes between the use of epoxy resin sealers and calcium silicate‐based sealers (Kangseng et al., 2025). However, the combined use of these materials and techniques, especially when combined with electronic apex locators and operating microscope, has not been extensively assessed in the literature.

Evaluating the effect of various treatment protocols on tooth survival and success of root canal treatment presents substantial challenges, due to the inherent limitations of retrospective studies, but even randomized controlled trials. Retrospective studies often lack detailed and accurate information about the specific treatment protocols used. This makes it difficult to draw reliable conclusions about their treatment effect, which are oftentimes complicated by the variability in clinical practices and documentation standards that may obscure critical factors influencing treatment outcomes (Talari & Goyal, 2020). On the other hand, while the randomized controlled trials provide more controlled and precise data, they are frequently limited by smaller sample sizes. These smaller patient cohorts can reduce the generalizability of the findings and limit the statistical power necessary to detect significant differences between treatment protocols (Bosdriesz et al., 2020; Saldanha et al., 2022), especially if the treatment methods used are expected to differ only slightly in terms of their success rate. The restrictions in both research designs create a major barrier to obtaining decisive information regarding how various treatment protocols affect the success of root canal treatment.

Non‐Surgical Root Canal Treatment (NSRCT) generally aims to preserve the natural dentition by eliminating diseases affecting the pulp and periapical tissues (Rotstein et al., 2006). The absence of apical periodontitis, in conjunction with sustained or restored clinical and radiographic normalcy, is commonly regarded as an indicator of a successful RCT procedure (Strindberg, 1956). Systematic reviews by Ng et al. (2007) and Ng, Mann, Rahbaran, et al. (2008) reported that the mean success rate, when strict criteria of periapical tissues evaluation were considered (complete absence of periapical radiolucency) was between 31% and 96% among the studies included. On the other hand, when more lenient criteria for success were considered, where the periapical radiolucency shrinks or remains the same size while all accompanying symptoms of inflammation disappear, the mean success rate increased to 60% to 100% in some studies. The authors of these studies continued to update their meta‐analyses using their published methods with new data until the end of 2020 (Gulabivala & Ng, 2023). The updated analyses demonstrated that 84% of vital teeth did not show signs of apical periodontitis after the treatment, while in other cases with preoperative periodontitis, the periapical lesion completely resolved in 74% of cases. However, other criteria for the success of the root canal treatment have been proposed, such as the functional survival of the treated tooth without signs or symptoms of inflammation (Farzaneh et al., 2004). Survival analyses as a type of success assessment have become more popular with the introduction of dental implants as a treatment alternative to RCT (Chatzopoulos et al., 2018). In addition, multistate analysis is a novel tool introduced in the fields of epidemiology and medical research (Matsena Zingoni et al., 2021). It allows studying the transitions between different health states or treatment interventions over time, which helps for a better understanding of disease progression, the efficiency of treatments and predicting patient outcomes in various medical fields (Jackson et al., 2022; Sundin et al., 2023).

The aim of this retrospective study was to evaluate tooth survival and tooth survival without further interventions of three different root canal treatment protocols, while also assessing various patient‐related and other clinical predictors that could potentially impact the success of RCT.

## MATERIALS AND METHODS

The present study was designed as an observational cohort study, wherein data collection was carried out retrospectively. The retrospective nature of data collection allowed for the study to be conducted without impinging upon the psychological or physical integrity of the patients. The study was carried out in compliance with the principles outlined in the declaration of Helsinki (World Medical Association, 2013) as well as following the Professional Code for Physicians of the Medical Council of the State of Schleswig‐Holstein, Germany. The study protocol received ethical approval from the Institutional Review Board (IRB No. D 495/24).

This publication was written following the ‘STrengthening the Reporting of Observational Studies (STROBE)’ guidelines (von Elm et al., 2007) and the ‘Preferred Reporting items for OBservational studies in Endodontics (PROBE)’ guidelines (Nagendrababu et al., 2023).

### Inclusion and exclusion criteria

All treatment cases where RCT was initiated between July 1999 and January 2024 were included. Teeth were excluded from the study if the treatment was incomplete, the patient was younger than 18 years old at the time of the treatment, or if teeth were treated surgically as the initial treatment. Deciduous and wisdom teeth were also excluded. Cases with incomplete records or missing variables were excluded as well.

### Data collection and acquisition of study samples

In this study, data analysis was carried out using a claims database that was specifically created for quality assurance reasons for the outpatient clinic of the Dental Academy of Continuing Professional Development in Karlsruhe, Germany. To ensure the privacy and confidentiality of the patients, all data were anonymized and extracted for analysis without any reference to individual patients. For this study, all data related to the teeth of the patients who started NSRCT at the Academy from July 1999 until January 2024 was included in the analysis. To facilitate data analysis, a programmed interface was developed that allowed for the extraction of tooth history information through a database query. The database utilized in this study employs a system of codes, that correspond to the ‘BEMA tariff’ (‘KZBV—Schedule of Fees’) of the statutory health insurance system in Germany. These codes encompass defined dental treatments administered to a given tooth. Additionally, the database includes invoice codes conforming to the tariff of private fees for dentists as outlined in the ‘Fee schedule for dentists “GOZ”’ (German Dental Association, 2023) established by the Federal Chamber of Dentists in Germany in 2019. The utilization of these codes enables a standardized means of organizing and tracking dental treatments.

In this database, cases that received RCT were identified by the code ‘WK’ which is the code for root canal preparation. After that, a history record was generated by conducting a database query for each identified case, which included all succeeding invoice codes along with their respective dates of service delivery, until the most recent interaction between a dentist and the tooth in question became available.

**FIGURE 1:**
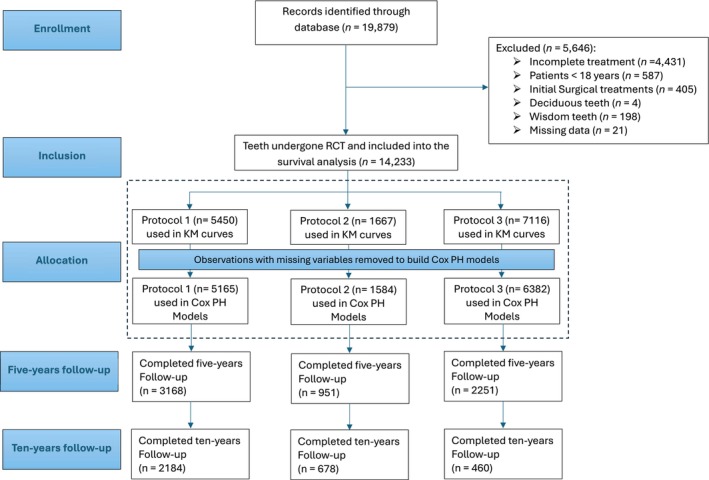
Flowchart for observations inclusion and exclusion criteria, allocation and numbers, with number of teeth at five‐year and ten‐year follow‐up. KM, Kaplan‐Meier; PH, Proportional hazards.

For the analysis, the date of root canal filling (‘WF’) was used as the starting point of the observation, excluding observations with incomplete treatments (see Figure 1) and pre‐planned surgical retreatment (codes ‘WR1’, ‘WR2’, ‘WR3’). The FDI Two‐Digit system was used to denote the treated teeth and to identify their type (anterior tooth, premolar or molar). It was also used to identify deciduous and wisdom teeth and exclude them from the analysis. Age at treatment was calculated from the patient's birth date to the ‘WF’ date. Treatments were classified as single‐visit if ‘WK’ date matched ‘WF’ date. Pulp status was determined by the codes ‘VitE’ for vital and ‘Trep1’ for non‐vital pulps. Patient gender, number of supportive periodontal treatments, total number of visits required to complete the RCT and previous root canal treatment in the same patient were directly extracted from the database. The treatment providers were identified by their ID number in the database, where all treatment providers were considered non‐experienced practitioners and only one was assigned as an experienced treatment provider. Regarding the type of RC sealer, in all treatments completed before 2020, epoxy resin‐based sealer was used, and after that Protocol 3 shifted to calcium silicate‐based sealer.

To ensure data completeness, incomplete records or cases with missing variables were excluded in the stage of data pre‐processing. In addition to that, any observation with missing data in the included covariates were excluded in the Cox proportional hazards regression analysis modelling process.

### Outcome measure

In this study, tooth survival without further interventions was considered as one outcome parameter. Further interventions included tooth extraction, surgical retreatment and non‐surgical retreatment of the tooth under observation, which were carried out only in cases where the treated tooth showed signs or symptoms of inflammation. The second outcome parameter was pure survival of the tooth, regardless of further interventions.

The demarcation of the endpoint of observation was set as one of four possibilities with their corresponding dates. First, when a tooth was extracted (invoice codes ‘X1’, ‘X2’, ‘X3’, ‘Ost1’ or ‘Ost2’), second, when surgical retreatment was performed (referred to as invoice codes ‘WR1’, ‘WR2’ or ‘WR3’), third, when another root canal preparation was performed on a date later than the date of root canal filling (indicated by invoice code ‘WK’ after the date of the first root canal filling ‘WF’), or the date of the last dentist‐patient contact which represents the last entry in the database when a dentist observed the tooth in place. In the case of the survival analysis until extraction, the endpoint of observation was the date of extraction or the date of the last dentist‐patient contact.

In the analysis, if no further interventions were recorded for a specific tooth until the end of our study, we marked the tooth as ‘censored’ at the date of last recorded visit, ensuring that the tooth was accounted for until its presence could no longer be confirmed. This approach aligns with standard practices in survival analysis (Cox, 1972). This approach ensured that we utilized all the available data and maintained accuracy by correctly handling censored cases.

### Endodontic treatment protocols

All endodontic treatments were performed by approved dentists with a corresponding degree. Only one of the operators had extensive experience in endodontics, while all other operators had no special endodontic training and they acted like general practitioners. All endodontic treatments were carried out following the guidelines and standards of good clinical endodontic practice (European Society of Endodontology, 2006), which includes the use of rubber dam in all included cases, regardless of the treatment provider and treatment protocol. The experienced treatment provider carried out all treatments under the operating microscope, while using the different treatment protocols as described below over time. Treatment protocols in the study groups have adapted to the technological advancements in root canal instrumentation that have taken place over the past decades. Three different protocols are documented:

#### Protocol 1 (*n* = 5450)

The RCT method adopted in this protocol was the standardized technique (Kerekes & Tronstad, 1979) using stainless steel hand K‐files (VDW, Munich, Germany). This protocol was used from July 1999 until 2007. The working length was established with the aid of radiographs. A minimum of a master apical file (MAF) of ISO 35 was achieved in all root canal preparations with a Taper of 0.02. Sodium Hypochlorite (NaOCl) 3.5% was used as a root canal irrigant to disinfect the canal using syringes and needles (Braun, Melsungen, Germany). After disinfection, the root canals were dried with paper points (Roeko Paper Points, Coltène/Whaledent GmbH + Co. KG, Langenau, Germany). In cases of multiple visits, freshly mixed calcium hydroxide from the pharmacy was used as an interappointment root canal dressing inserted into the root canal with a lentulo spiral ISO 25 (VDW, Munich, Germany). The obturation of the root canals was done by the cold lateral compaction technique using gutta‐percha (Roeko Guttapercha Points 0.02 Taper, Coltène/Whaledent GmbH + Co. KG, Langenau, Germany) and epoxy resin‐based root canal sealer (AH Plus, Dentsply, Konstanz, Germany). The sealer was inserted into the root canal with the master point. The cold lateral compaction was done with finger spreaders ISO 25 (VDW, Munich, Germany) and the placement of accessory gutta‐percha points.

#### Protocol 2 (*n* = 1667)

In the year 2008, new techniques and instruments were added to the outpatient clinic armamentarium for endodontic treatments. These included rotary NiTi instruments and Passive Ultrasonic Irrigation (PUI). RaCe and BioRaCe rotary instruments (FKG, La Chaux de Fonds, Switzerland) were used for the canal preparation. The apical size of preparation was at least ISO 35 and a taper of 0.04. Before the rotary preparations, a manual glide path preparation was done using stainless steel hand instruments up to a #15 K‐file. The working length of the root canal was determined using apex locators and radiographs. The irrigation solutions used were 3.5% NaOCl and 15% citric acid, with occasional addition of 2% chlorhexidine. After the mechanical preparation, PUI was applied using either an ultrasonic‐activated spreader or an IRRI‐K file (Acteon, Mettmann, Germany) with an ultrasonic device. After disinfection, the root canals were dried with paper points (Roeko Paper Points, Coltène/Whaledent GmbH + Co. KG, Langenau, Germany). In cases of multiple visits, fresh mixed calcium hydroxide was used as interappointment root canal dressing. The root canals were obturated with cold lateral compaction of Gutta‐percha and AH Plus as described in the first protocol. This method was adopted until November 2011.

#### Protocol 3 (*n* = 7116)

Commencing from November 2011, the endodontic procedures were exclusively conducted using Reciproc instruments (R25, R40 and R50, VDW, Munich, Germany) in compliance with the manufacturer's instructions for glide path‐free preparation (VDW Dental, 2015). In a few situations when Reciproc instruments were not able to reach full working length, the glide path was then prepared manually. The apical master file was at least the R25, resulting in an apical preparation size of ISO 25 with a taper of 0.08. The determination of working length was achieved with the apex locator. Additionally, radiographs were used in cases where the treatment provider deemed them necessary (e.g. implausible behaviour of the apex locator). The disinfection of the root canal was consistently conducted by utilizing PUI, and the smear layer was removed through the application of 18% Ethylenediaminetetraacetic acid (EDTA). Final root canal disinfection was achieved using NaOCl 3.5%. After disinfection, the root canals were dried with paper points (Reciproc paper points, VDW, Munich, Germany). In cases of multiple visits, fresh mixed calcium hydroxide was used as interappointment root canal dressing. Finally, the root canal obturation was performed either by lateral compaction or warm vertical compaction, using gutta‐percha and epoxy resin‐based sealer (AH Plus, Dentsply, Konstanz, Germany). From June 2020 on, calcium silicate‐based root canal sealer (BioRootRCS, Septodont, Niederkassel, Germany) was used. In cases of cold lateral compaction, the method was the same as in Protocol 1. Only the master cones were changed to system gutta‐percha (Reciproc gutta‐percha, VDW, Munich, Germany). In cases of warm vertical compaction, the continuous wave technique (Buchanan, 1996) was used. A heat plugger (B&L Superendo alpha II, B&L Biotech inc., Fairfax, USA) was used for the downpack of system gutta‐percha (Reciproc gutta‐percha, VDW), while the backfill was accomplished with a gutta‐percha obturation gun (B&L Superendo beta II, loaded with B&L Gutta Percha Pellets soft, both B&L Biotech inc.).

To separate the previously mentioned treatment protocols from each other, the ‘Phys’ invoice code was utilized, as all treatments that did not include this code were performed by Protocol 1. For all other observations where ‘Phys’ code was detected, the treatment belonged to the multiple file systems Race/BioRace (FKG, La Chaux de Fonds, Switzerland) if it was performed up to October 2011. After this date, all treatments were strictly performed by Protocol 3 supplemented with PUI.

In this study, we aimed to evaluate the influence of these three treatment protocols and other factors on pure tooth survival until extraction and tooth survival until the first further intervention (which includes surgical and non‐surgical retreatment and tooth extraction). In the latter assessment, the exact sequence of interventions cannot be addressed. For this reason, we assessed the progression of treatment interventions among different treatment states using a multistate model. Additionally, to account for confounders and groups imbalance, we achieved balance in baseline characteristics between treatment protocols utilizing two statistical methods: Entropy balancing and Generalized Boosted Logistic Regression (GBLR).

### Statistical analysis

For the statistical application and data manipulation in this study, R (R version 4.2.2, R Foundation for Statistical Computing, Vienna, Austria) was used with RStudio (R Core Team, 2024) using R‐scripts written solely for this purpose.

For the survival analyses, tooth survival was either calculated until the first further intervention or until extraction. In case of no events, survival was calculated until the last dentist‐patient contact for censoring. Then, the Kaplan–Meier curve was plotted using the survival R package (Therneau et al., 2023) for each of the treatment protocols separately. In addition to that, Cox proportional hazard regression was built to investigate the effect of the type of treatment protocol together with other potential factors on tooth survival or tooth survival without further intervention including the age (which was analysed as a continuous variable in the statistical models) and gender of the patient, experience of the treatment provider, tooth type, number of supportive periodontal treatments, number of visits of RCT, number of previous RCT, type of RC sealer and preoperative tooth vitality. In the first step, a separate Cox proportional hazards model for each of the variables was calculated, and in the second phase, all the variables were added to the model simultaneously. The level of significance was set at *p* = .05. Teeth in place without recorded further interventions by the last dentist‐patient contact were treated as right‐censored observations in the Cox proportional hazards regression analysis. This method ensures that censored cases contribute to the risk set until their last follow‐up date, without assuming indefinite survival beyond this point. To address potential confounding factors and improve the balance of the treatment protocols, entropy balancing (EB) was performed using the WeightIt R Package (Greifer, 2024), where weights are assigned to each of the observations to attain this aim without excluding any of them. This balancing approach ensured that the protocols were more similar in terms of their baseline characteristics. The weights obtained from EB were then used in a second Kaplan–Meier curve and Cox proportional hazard regression analysis. We used another balancing approach for the covariate balancing, which was Gradient Boosted Logistic Regression (GBLR) using the Twang R package (Griffin et al., 2023), in which weights were assigned to observations and used to build a new Kaplan–Meier curve and Cox proportional hazard regression analysis.

**FIGURE 2:**
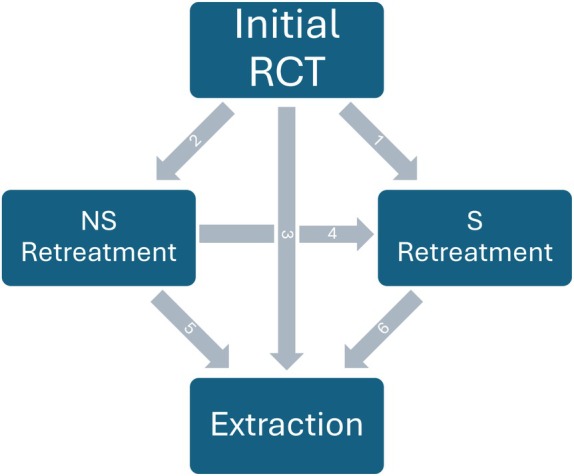
The six possible transitions between the four intervention states in the multistate analysis: Initial RCT (root canal treatment) or no intervention, NS (non‐surgical) retreatment, S (surgical) retreatment and extraction.

With these types of survival analyses, it is not possible to estimate the effect of variables on the incidence of each intervention separately. Moreover, the analysis does not follow up the treated teeth after the first intervention was recorded. Hence, in order to evaluate the influence of the protocols on the incidence of retreatment and extraction separately and to follow up teeth until their last intervention (extraction) we also built a multistate analysis model using the mstate R package (de Wreede et al., 2011) to try to estimate the effect of different treatment protocols on the probability of a transition between different tooth intervention states (no intervention, non‐surgical retreatment, surgical retreatment and extraction, see Figure 2). Cox proportional hazards regression, using stratification by treatment protocol, was implemented to assess whether the transitions are affected by the treatment protocol or not.

## RESULTS

### Study population characteristics

A total of 19 879 teeth were identified in the period from January 1999 to January 2024, where root canal treatment was initiated. From these cases, 4431 were excluded (Figure 1) because the root canal treatment was not completed. 587 cases were excluded because patients were not yet of the age of 18 at the time of the treatment, and 405 cases were excluded because they underwent surgical RCT on the same day of treatment. 4 deciduous teeth, 198 wisdom teeth, and 21 other cases with implausible entries or missing documentation were also excluded from the data analyses. The total number of teeth included in the analyses was 14 233.

### Baseline characteristics

Table 1 shows the number of teeth in every treatment protocol together with baseline characteristics of all observations. During a period of nearly 25 years of follow‐up, the number of teeth without recorded further interventions was 11 199 cases (79%). Extraction was the most common intervention (*n =* 2.359, 17% of total cases) followed by non‐surgical retreatment (*n* = 378, 2.7% of total cases), while surgical retreatment was the least common intervention (*n* = 297, 2.1% of total cases). Treatment Protocols 2 and 3 were exceptions to this routine as surgical retreatment was implemented slightly more often than non‐surgical retreatment. The need for further interventions in treatment Protocols 1, 2 and 3 was 32.3%, 28.1% and 11.2%, respectively, if the observation time is disregarded.

**TABLE 1:** Baseline characteristics and fate of the observations.

Characteristic	Overall	Protocol 1	Protocol 2	Protocol 3	*p* value^a^
	*N* = 14 233	*N* = 5450	*N* = 1667	*N* = 7116	
Number of visits for RCT, *n*/*N* (%)					<.001
Multiple	9737/14 233 (68%)	4698/5450 (86%)	1561/1667 (94%)	3478/7116 (49%)	
Single	4496/14 233 (32%)	752/5450 (14%)	106/1667 (6.4%)	3638/7116 (51%)	
Patient age, mean (SD)	52.87 (16.27)	51.36 (16.48)	51.80 (16.51)	54.27 (15.92)	<.001
Pulp vitality, *n*/*N* (%)					<.001
Nonvital	7800/13 139 (59%)	2670/5168 (52%)	849/1585 (54%)	4281/6386 (67%)	
Vital	5339/13 139 (41%)	2498/5168 (48%)	736/1585 (46%)	2105/6386 (33%)	
(missing)	1094	282	82	730	
Supportive periodontal treatments, mean (SD)	2.81 (5.54)	2.65 (6.00)	4.63 (6.98)	2.50 (4.63)	<.001
Type of further intervention, *n*/*N* (%)					<.001
Eventless survival	11 199/14 233 (79%)	3674/5450 (67%)	1202/1667 (72%)	6323/7116 (89%)	
Non‐surgical retreatment	378/14 233 (2.7%)	274/5450 (5.0%)	39/1667 (2.3%)	65/7116 (0.9%)	
Surgical retreatment	297/14 233 (2.1%)	182/5450 (3.3%)	46/1667 (2.8%)	69/7116 (1.0%)	
Extraction	2359/14 233 (17%)	1320/5450 (24%)	380/1667 (23%)	659/7116 (9.3%)	
Gender, *n*/*N* (%)					.008
Female	7269/14 224 (51%)	2715/5447 (50%)	827/1666 (50%)	3727/7111 (52%)	
Male	6955/14 224 (49%)	2732/5447 (50%)	839/1666 (50%)	3384/7111 (48%)	
(missing)	9	3	1	5	
Tooth type, *n*/*N* (%)					<.001
Anterior tooth	3550/14 233 (25%)	1537/5450 (28%)	397/1667 (24%)	1616/7116 (23%)	
Premolar	4286/14 233 (30%)	1738/5450 (32%)	539/1667 (32%)	2009/7116 (28%)	
Molar	6397/14 233 (45%)	2175/5450 (40%)	731/1667 (44%)	3491/7116 (49%)	
Treatment provider, *n*/*N* (%)					<.001
Experienced dentist	3523/14 233 (25%)	253/5450 (4.6%)	455/1667 (27%)	2815/7116 (40%)	
General practitioner	10 710/14 233 (75%)	5197/5450 (95%)	1212/1667 (73%)	4301/7116 (60%)	
Number of visits (by number), mean (SD)	2.20 (1.23)	2.80 (1.42)	2.61 (0.95)	1.64 (0.80)	<.001
Previous RCTs, mean (SD)	0.71 (1.15)	0.63 (1.02)	0.77 (1.12)	0.76 (1.25)	<.001
Time before obturation, mean (SD)	41.39 (131.65)	57.08 (151.65)	60.08 (231.82)	25.00 (62.70)	<.001
RC sealer, *n*/*N* (%)					<.001
Calcium silicate‐based	1975/14 233 (14%)	0/5450 (0%)	0/1667 (0%)	1975/7116 (28%)	
Epoxy resin‐based	12 258/14 233 (86%)	5450/5450 (100%)	1667/1667 (100%)	5141/7116 (72%)	

^a^ Pearson's Chi‐squared test; Kruskal–Wallis rank sum test.

The number of further interventions by the ten‐year landmark was 2650 observations, 1463 (26.8%), 405 (24.2%) and 782 (11%) for the three treatment protocols, respectively. While 15 years after the initial treatment, 2926 teeth were retreated or extracted (1668 (30.6%), 465 (27.9%) for the treatment Protocols 1 and 2, respectively). The survival probability of teeth from the three protocols is shown in Table 2. During the period of ten to 14 years after treatment, there was only a 0.1% increase in the need for further interventions in Protocol 3.

**TABLE 2:** Survival probability of the three protocols at different timelines.

Years	Probability of survival		
	Protocol 1	Protocol 2	Protocol 3
At 1 year	0.929	0.935	0.963
At 5 years	0.757	0.775	0.832
At 10 years	0.59	0.602	0.688
At 15 years	0.49	0.452	NA
At 20 years	0.394	NA	NA

### Accounting for group imbalances

Before advancing to further analyses, an assessment of covariate balance across the three treatment protocol groups was performed, uncovering imbalances in their distribution. To address this issue, two statistical approaches were employed for group balancing: EB and GBLR, the latter is a method under the umbrella of Generalized Boosted Models (GBM). These methods assigned weights to each observation, without excluding any of them, based on their baseline characteristics, subsequently used in Cox proportional hazard regression analysis and constructing Kaplan–Meier curves. Covariate balance was reassessed post‐weighting, revealing equilibrium between the protocols. The balance obtained from EB was better compared to the balance obtained from GBLR.

### Survival analysis

**FIGURE 3:**
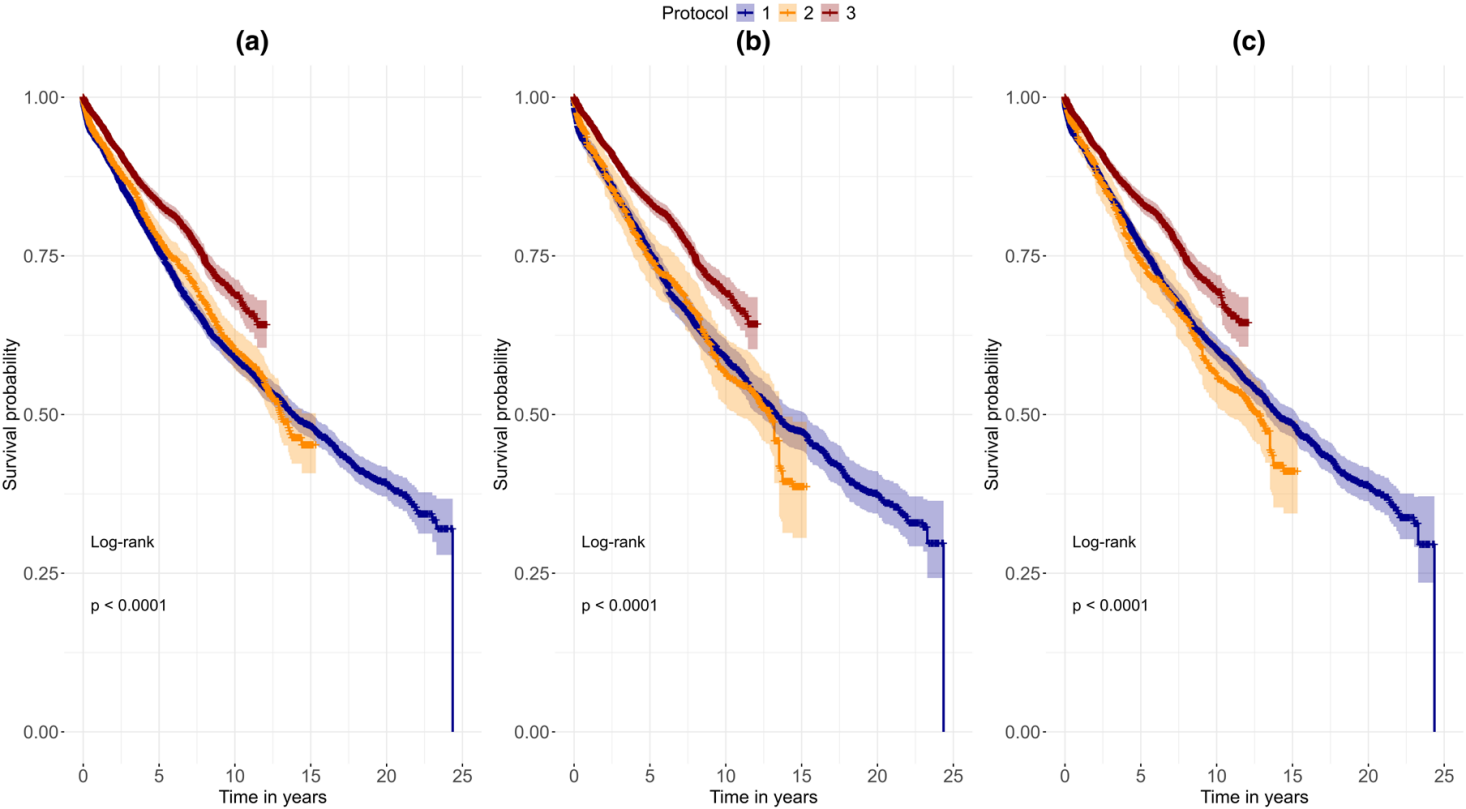
Kaplan–Meier curves for survival until the first further intervention. (a) Shows the survival without group balancing, (b) shows survival with Entropy Balancing and (c) shows survival with Gradient Boosted Logistic Regression balancing. Protocol 3 shows significantly higher probability of survival when compared to the other protocols.

**FIGURE 4:**
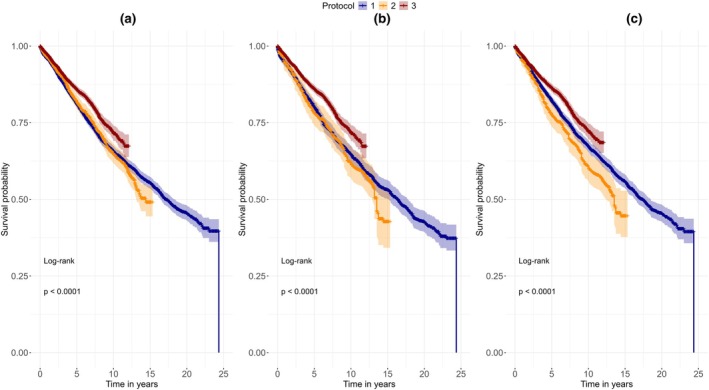
Kaplan–Meier curves for survival until tooth extraction. (a) Shows survival without group balancing, (b) shows survival with Entropy Balancing, and (c) shows survival with Gradient Boosted Logistic Regression balancing. Protocol 3 shows significantly higher probability of survival when compared to the other protocols.

The mean observation time for the study sample was 5.84 years (min. = 0.0, max = 24.56 years). Kaplan–Meier curves were plotted for the analysis with and without group balancing with EB and GBLR for survival until the first further intervention and for pure survival until extraction (Figures 3 and 4). Protocol 3 consistently exhibits a higher probability of survival across all plots compared to the other treatment protocols. At the landmark of ten years after initial treatment, the probability of survival of Protocols 1, 2 and 3 was 59.0%, 60.2% and 68.8%, respectively (Table 2). We also observed that the probability of tooth survival in Protocol 2 was marginally greater than that of Protocol 1 in the first few years following the initial treatment, which is reversed after 11 to 12 years (at 15 years landmark probability of survival of Protocol 1 and 2 was 49.0% and 45.2%, respectively).

**FIGURE 5:**
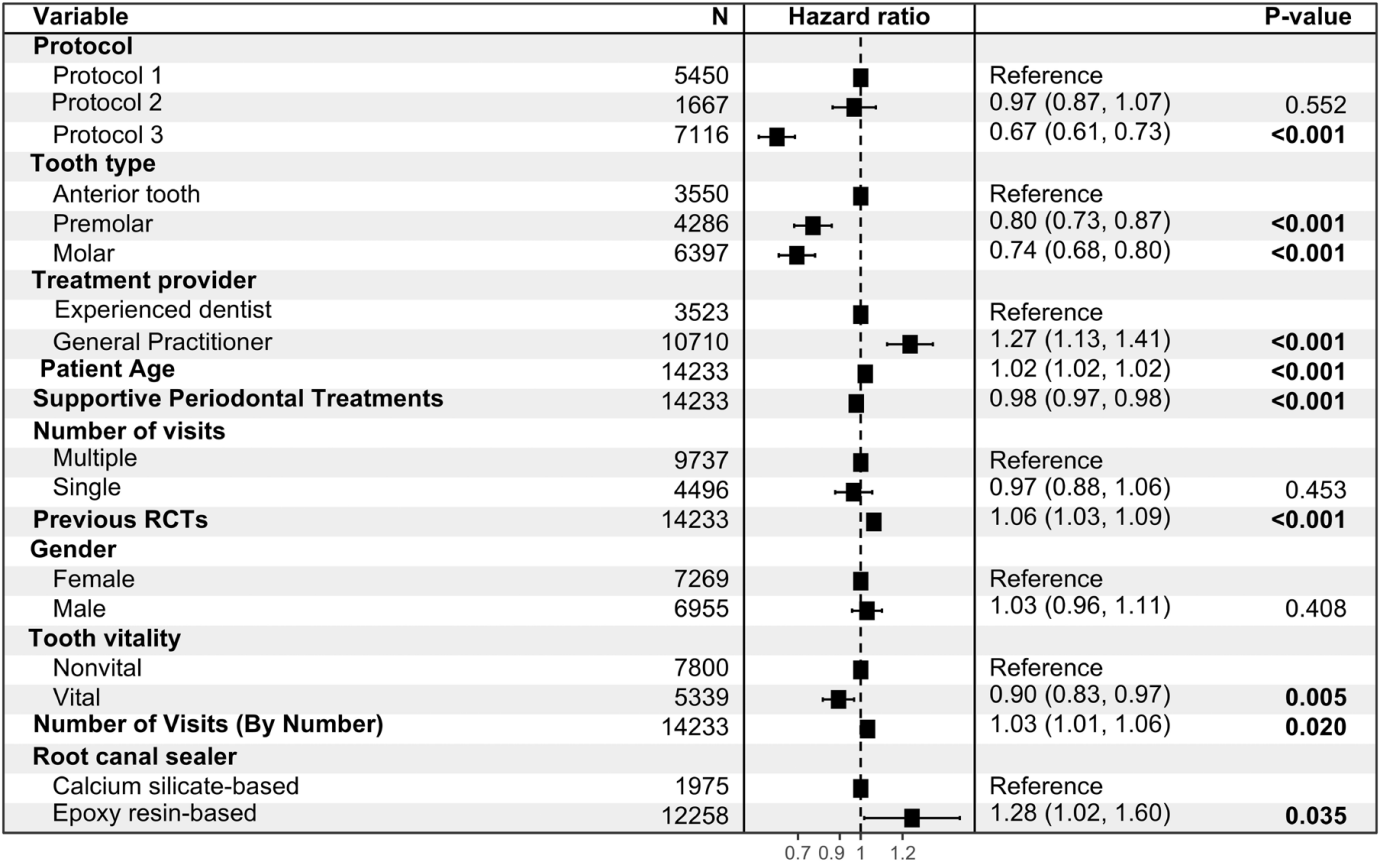
Forest plot of the Cox proportional hazards regression of the unadjusted estimates of the included covariates.

**FIGURE 6:**
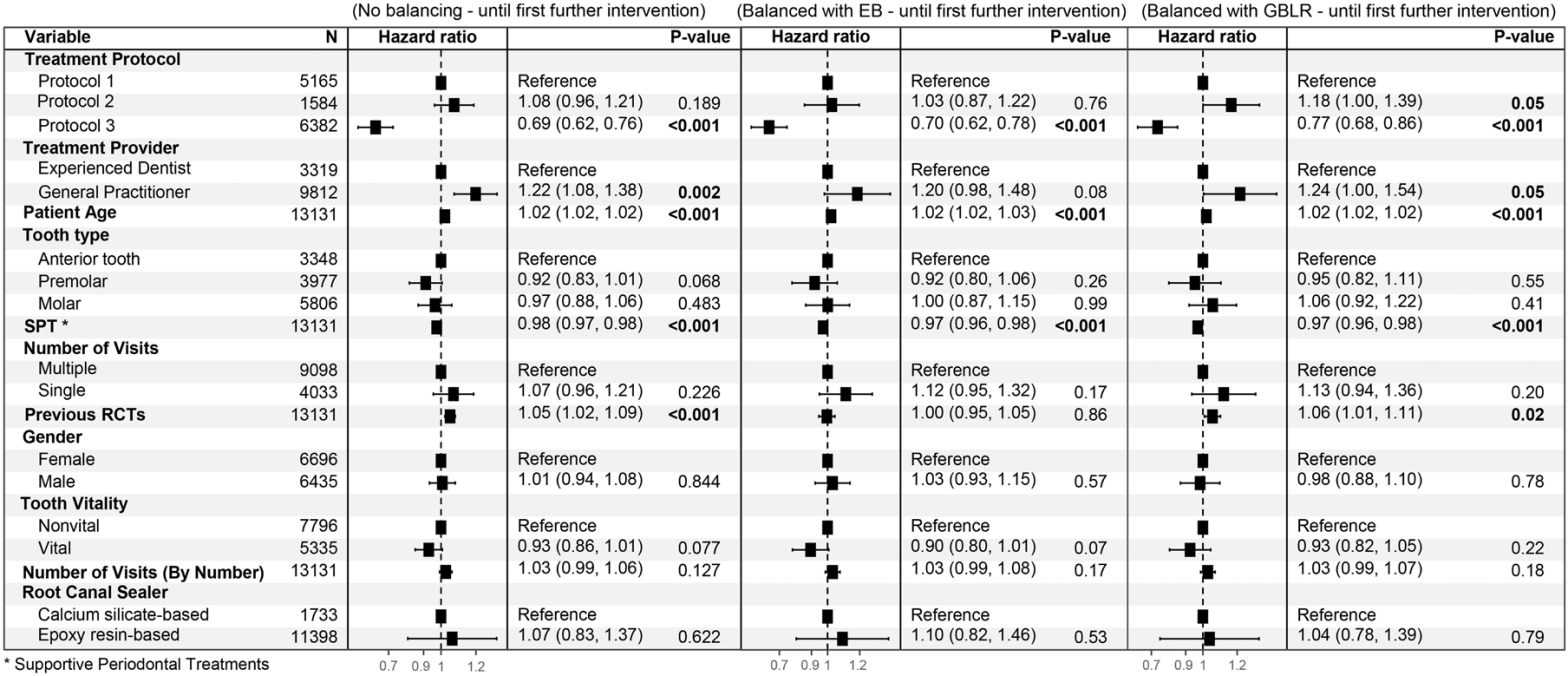
Forest plots of the Cox proportional hazards regression models of survival until the first further intervention. The first plot on the left shows the results for the unbalanced groups, the middle plot shows the results with Entropy Balancing (EB) and the right plot shows the results with Gradient Boosted Logistic Regression (GBLR) balancing. In all models, Protocol 3 shows lower hazard ratios (HR) compared to the other protocols. Patient age and supporting periodontal treatments (SPT) are also seen to significantly affect the results across all models, with narrow confidence intervals (CI). No. visits, number of visits; SPT, supporting periodontal treatments.

**FIGURE 7:**
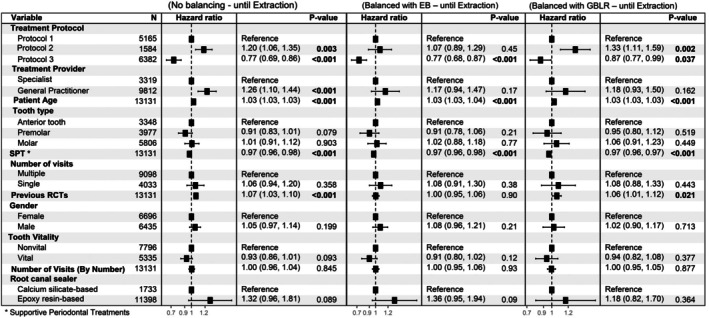
Forest plots of the Cox proportional hazards regression models of survival until tooth extraction. The first plot on the left shows the results for the unbalanced groups, the middle plot shows the results with EB, while the plot on the right exhibits the results of the model with GBLR balancing. In all models, Protocol 3 shows lower hazard ratios (HR) compared to the other protocols. Patient age and supporting periodontal treatments (SPT) are also seen to significantly affect the results across all models with narrow confidence intervals (CI). No. visits, number of visits; SPT, supporting periodontal treatments.

In the Cox proportional hazard regression analysis with unadjusted estimates, treatment protocol, tooth type, experience of the treatment provider, patient age, number of supportive periodontal treatments, pre‐treatment tooth vitality, type of RC sealer and number of visits to complete the RCT were all found to have a statistically significant effect on the outcome (Figure 5). However, few of these variables (treatment protocol, experience of the treatment provider, patient age, number of supportive periodontal treatments) retained statistical significance after adjusting for other variables (Figures 6 and 7). In all models, Protocol 3 consistently demonstrated significantly lower cumulative hazards compared to the first treatment protocol (*p* < .001). Following the adjustment of weights to address covariate imbalances using both balancing techniques, Protocol 3 maintained the significantly lower cumulative hazard. The outcome also revealed that each year increase in patient age at the time of treatment increases the cumulative hazard by 2% per year (*p* < .001). Besides patient age, an increase in the number of performed supportive periodontal treatments (SPT) significantly decreases the hazard ratio (*p* < .001 across all models). Experience of the treatment provider affects the outcome as cases provided by a general practitioner exhibited a significantly higher cumulative hazard (*p* = or <.001 for models without covariate balancing). The number of prior root canal treatments received by the patient similarly affected the outcome (*p* = or <.001 for models without covariate balancing). Effects of the latter two variables were not significant when the group balancing methods were applied to the analysis, specifically using EB. On the other hand, tooth type, tooth vitality, whether the treatment was performed in single or multiple visits, gender of the patient, and the overall number of visits required to finish the RCT had no statistically significant influence on the outcome throughout all analyses. Finally, the multistate Cox model was built to examine the effect of the treatment protocol on the transitions between different intervention states. Six different transitions were considered in this study (Figure 2). Table 3 shows the incidence of all transitions for the three treatment protocols. The table shows that most transitions after the initial treatment were directed toward extraction (*n* = 2359). Nevertheless, the treatment protocols differ regarding which transition comes next. While for Protocol 1, the second most common transition is the transition from initial treatment to non‐surgical retreatment (*n* = 274), for Protocol 2 and Protocol 3 the transition from initial treatment to surgical retreatment is the second most common (*n* = 46 and *n* = 69 for the two protocols, respectively, Table 3).

**TABLE 3:** Transitions between intervention states by number in treatment protocols.

Transition	Protocol 1	Protocol 2	Protocol 3
Initial treatment to non‐surgical retreatment	274	39	65
Initial treatment to surgical retreatment	182	46	69
Initial treatment to extraction	1320	380	659
Non‐surgical retreatment to surgical retreatment	15	2	2
Non‐surgical retreatment to extraction	95	15	11
Surgical retreatment to extraction	86	13	10

**FIGURE 8:**
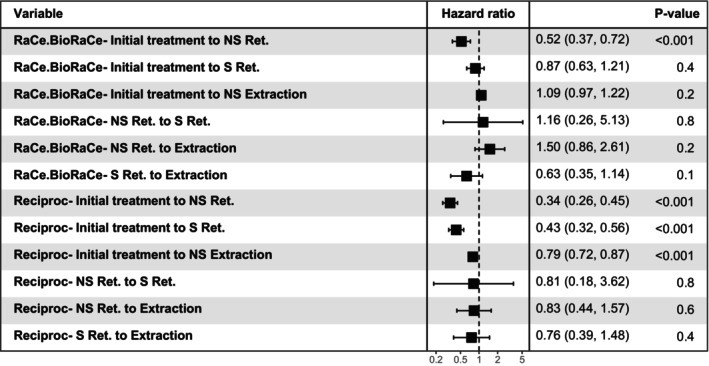
Forest plot showing the hazard ratio (HR) with confidence intervals (CI) for the multistate model with the six different transitions comparing the three treatment protocols. Note that the reference for each transition is Protocol 1 (hand files protocol). NS Ret, non‐surgical retreatment; S Ret., surgical retreatment.

On the application of Cox proportional hazards regression, using stratification by treatment protocol, Protocol 3 reduced drastically the incidence of non‐surgical retreatment after the initial treatment (HR 0.34 (95% CI [0.26, 0.45]), *p* < .001), as well as the incidence of surgical retreatment and extraction after the initial RCT (HR 0.43 (95% CI [0.32, 0.56]), *p* < .001, HR 0.79 (95% CI [0.72, 0.87]), *p* < .001, respectively). The differences between Protocols 1 and 3 for the remaining transitions were not significant. On the other hand, Protocol 2 demonstrated a statistically significant reduction in non‐surgical retreatment compared to Protocol 1, while differences in other transitions remained insignificant (Figure 8).

**FIGURE 9:**
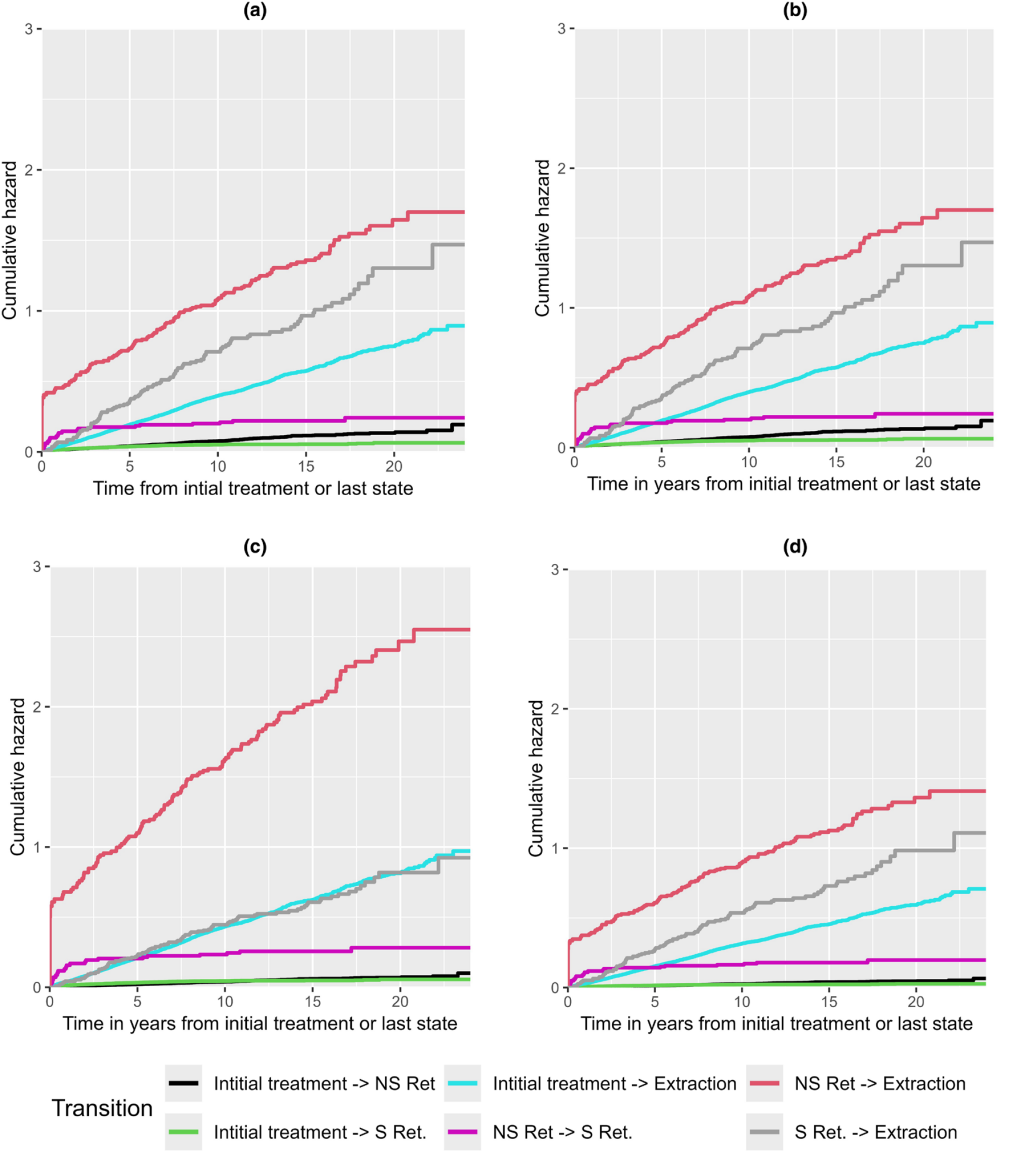
Cumulative Hazards of the incidence of transitions between intervention states; Initial treatment or no intervention, NS Ret (non‐surgical retreatment), S Ret (surgical retreatment) and extraction as an absorbing state. (a) Shows overall cumulative hazards for all observations. (b) Exhibits cumulative hazards of observations in Protocol 1. (c) Shows cumulative hazards of observations in Protocol 2. (d) Shows cumulative hazards of observations in Protocol 3.

**FIGURE 10:**
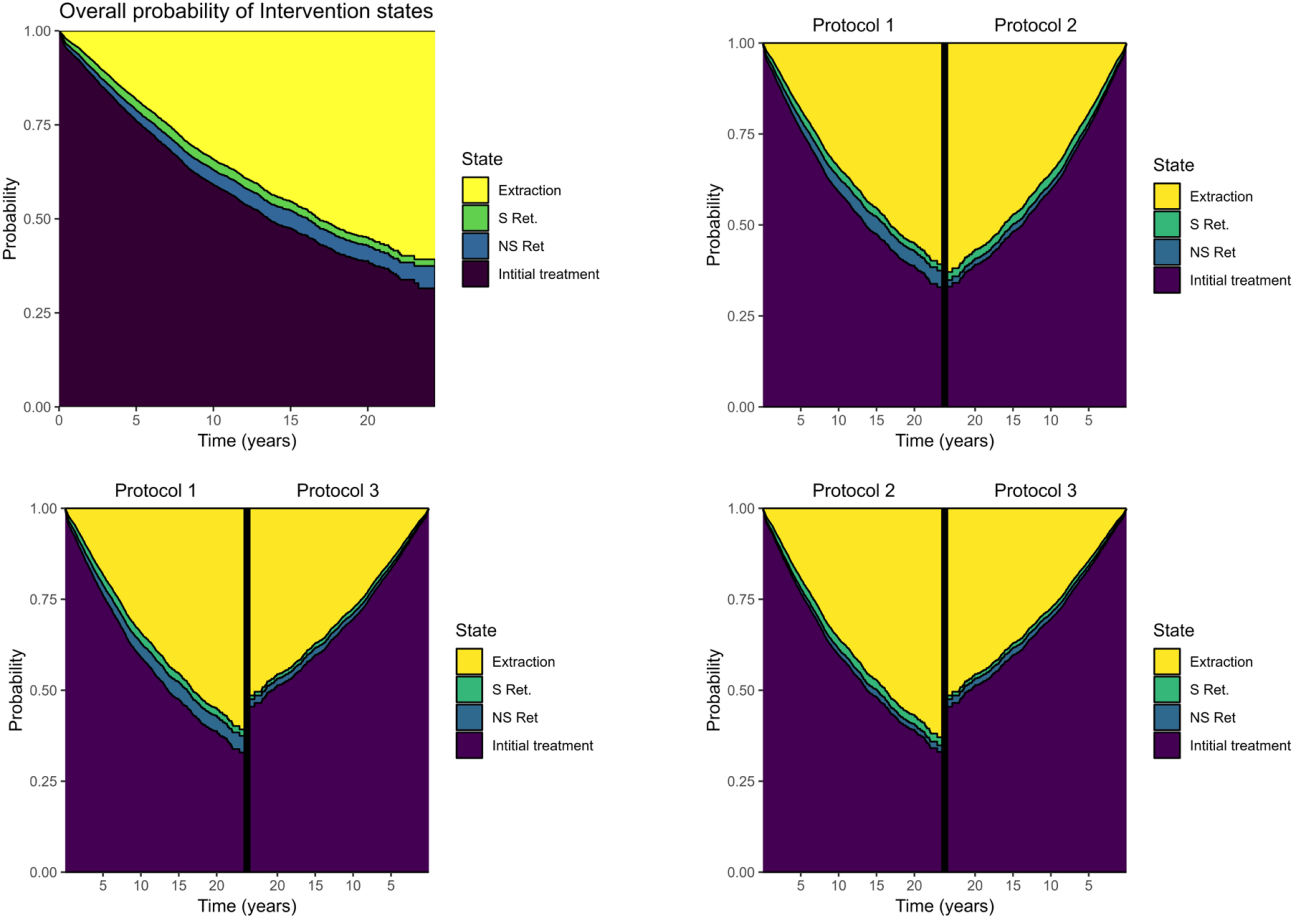
The probability plots show the probability of teeth being at different intervention states over time (Initial treatment or no intervention, NS Ret (non‐surgical retreatment), S Ret (surgical retreatment) and extraction as an absorbing state), for all the observations together and comparing the three treatment protocols. The plots demonstrate clearly that the probability of teeth receiving no further interventions is higher in Protocol 3 than in the other two treatment protocols.

Upon plotting the cumulative hazard for the transitions for all observations over a period of 24 years, and the treatment protocols separately (Figure 9), we observed that teeth were more likely to undergo extraction than to be retreated surgically or non‐surgically. The data also pointed out that during the short period after the tooth has been non‐surgically retreated, there was a high incidence of extractions. Figure 9 highlights that the incidence of transitions within Protocol 3 is notably lower compared to the other protocols. Figure 10 illustrates the probability of teeth being in a specific intervention state across all observations over the period of 24 years, with comparisons between treatment protocols. It remains clear with these probability curves that the probability of teeth staying without further interventions in Protocol 3 is higher than Protocol 1 and 2.

A thorough bootstrap sensitivity analysis was carried out in our investigation to assess the stability of our Cox proportional hazards model (Platt et al., 2000). We evaluated the variability of model coefficients through resampling from the original dataset using the boot package (Canty & Ripley, 2021) in R, providing a glimpse into the possible impact of various data subsets on our findings. Our results indicate the stability of our model by showing small bias (maximum bias 0.00122) and low standard errors (maximum standard error 0.061) in the calculated coefficients. We strengthened the validity of our study findings and the reliability of our statistical conclusions by implementing bootstrap sensitivity testing into our analytical framework. To further check the reliability of the analysis, we examined the interaction between the treatment protocol and various other factors. For the majority of the variables, our results showed no significant interaction. However, the relationship between molars as a tooth type and Protocol 3 was a significant exception (HR 0.71 (95% CI [0.58, 0.88]), *p* = .002). This suggests that the use of the Reciproc system for molar teeth was linked to a 28.63% reduction in hazard ratio in comparison to the reference group, which in this instance is the anterior teeth. These results were adjusted for other variables in the model.

## DISCUSSION

In this retrospective study, the long‐term survival of root canal‐treated teeth was evaluated using three different protocols: Protocol 1 (utilizing hand files, syringe irrigation with sodium hypochlorite and cold lateral compaction), Protocol 2 (utilizing rotary NiTi systems, adding apex locators, passive ultrasonic irrigation [PUI], citric acid and chlorhexidine) and Protocol 3 (Reciproc instruments adding apex locators, passive ultrasonic irrigation [PUI], EDTA, warm vertical obturation and calcium silicate‐based sealer). The findings of this study demonstrate that Protocol 3 significantly outperformed both Protocols 1 and 2 in terms of tooth survival without further intervention as well as overall tooth survival across all measures over a long observation period of more than 13 years and a large sample size. To the best of our knowledge, this is the first study to utilize entropy balancing (EB) and generalized boosted models (GBM) to enhance the reliability of results, and Protocol 3 retained its superiority even after adjusting for potential confounding variables. These findings call into question the prevailing notion that treatment protocols or instrumentation types may only have a minimal or no impact on endodontic outcomes, underscoring the enhanced efficacy and effectiveness of Protocol 3. Furthermore, the survival analysis demonstrated that, in the initial follow‐up period, Protocol 2 exhibited superior outcomes in comparison to Protocol 1. However, this trend reversed approximately a decade after the initial treatment, which is consistent with the findings of Mareschi et al. (2020), who reported that a protocol incorporating rotary NiTi files was more effective than hand files at the beginning of the follow‐up period, but that there was no significant difference at five years after treatment. Conversely, earlier studies had noted generally higher failure rates with hand files compared to rotary files (Cheung & Liu, 2009; Fleming et al., 2010). The initial success of rotary NiTi files might be attributed to their superior shaping ability, reduced debris extrusion, shorter instrumentation time and lower procedural errors (Bergmans et al., 2001; Nouroloyouni et al., 2023). Compared to both other treatment protocols, Protocol 3 showed a 30% reduction in the need for further interventions. Furthermore, the results of the multistate analysis demonstrated that it also led to a 66% reduction in the incidence of non‐surgical retreatment. The relevant question remains as to what the better performance of Protocol 3 is due to. In principle, the use of the Reciproc system, but also potentially the additional armamentarium used during the therapy, could be considered. This armamentarium encompasses apex locators, PUI, EDTA, warm vertical condensation obturation techniques and calcium silicate‐based RC sealers. However, the retrospective nature of the study design precluded the inclusion of some of the abovementioned potential cofactors in the analysis, rendering it impossible to reach a definitive conclusion as to whether one single factor had the biggest impact leading to Protocol 3's superiority. Nevertheless, it is worthwhile to consider and deliberate on which of these factors could exert the biggest impact.

A recent umbrella review concluded that the methods using apex locators or radiographs to determine the working length are equally valid regarding clinical outcomes (Pisano et al., 2024). A further systematic review and meta‐analysis determined that the generation of the apex locator does not influence the accuracy of the device in determining the working length (Nasiri & Wrbas, 2022). In our study, apex locators have been incorporated in both Protocols 2 and 3, yet Protocol 3 has been shown to surpass Protocol 2 in all models. This also applies to PUI. The latter factor has also been found to play a non‐significant role in periapical healing (Liang et al., 2013; Silva‐Filho et al., 2024) and therefore might also have a questionable impact at all. At least PUI was used in Protocols 2 and 3, but not in Protocol 1 and therefore cannot explain the superiority of Protocol 3 alone.

Citric acid and EDTA have been utilized in this study as an adjunct to sodium hypochlorite and PUI in Protocols 2 and 3, respectively. The findings of Ng et al. (2011a, 2011b) have indicated that the utilization of EDTA has been shown to increase the odds of periapical healing of root canal‐treated teeth by 1.3 to 2.3. However, preliminary research indicated that both methods are equally effective in removing the smear layer to allow better penetration of disinfectants and sealants into the dentinal tubules and enhance apical and coronal sealing ability. For instance, Kaushal et al. (2020) concluded in their study that both solutions are equally effective in removing the smear layer from the coronal and middle third of the canal, but that 10% citric acid was even more effective in the apical third. These findings are consistent with those of another study that compared the penetration of calcium silicate‐based sealer into dentinal tubules after 17% EDTA, 10% citric acid and 7% maleic acid (Shekhar et al., 2023). The study found that the three solutions were equally effective in the coronal third. However, citric and maleic acids were found to be more effective in penetrating the sealer in the middle and apical thirds. As demonstrated in the study by Sceiza et al. (2001), citric acid has been shown to possess reduced cytotoxic properties in comparison to EDTA when administered over shorter periods of time. Given that Protocol 2 utilized citric acid and Protocol 3 employed EDTA, the comparable properties of these materials imply that this factor exerted minimal influence on the observed discrepancies between the two protocol groups. In Protocol 2, Chlorhexidine has sometimes been used as an additional irrigant. However, it has been suggested in literature that it negatively affects the outcome of the treatment (Ng et al., 2011a, 2011b). If it is therefore assumed that the use of EDTA or citric acid is not decisive for the success of the treatment, chlorhexidine remains as a potential factor that cancels out the potential advantage of the rotary instruments in Protocol 2.

In addition to this, a systematic review by Pirani and Camilleri (2023)—despite acknowledging high bias in the studies included—found that obturation techniques, whether cold or warm compaction and materials used generally did not affect the healing of apical periodontitis. Given that Protocol 3 employed warm vertical compaction and Protocols 1 and 2 relied on cold lateral compaction, the equal effectiveness of the two methods suggests that this factor likely played only a minor influence in the observed variations between the groups. In 2020, calcium silicate‐based sealer was incorporated into Protocol 3, and it was hypothesized that this would offer a distinct advantage over the other protocols. A substantial body of research has been published on the subject, and it has been demonstrated that calcium silicate‐based sealers have a number of advantages over epoxy resin‐based sealers, including higher biocompatibility, bioactivity and antimicrobial activity (Donnermeyer et al., 2019; Lim et al., 2020). However, despite these advantages, the influence of calcium silicate‐based sealers on the outcome of RCT has not been actually proven (Kangseng et al., 2025), which agrees with the findings of our study, where the calcium silicate‐based sealer was not a significant factor in the adjusted models.

It seems that factors such as the use of apex locators, irrigation solutions, obturation methods and sealers are perhaps not the main reasons for the variations in outcomes across the three treatment protocols. While we cannot be entirely certain, the findings give a hint that the superior performance of Protocol 3 might be attributable to the specific features of the Reciproc system. In this context, it is important to note that achieving patency at the canal terminus has been found to increase the odds of success substantially, by more than twofold (Ng et al., 2011a). In contrast, the odds of success decrease by 12% for every one millimetre of ‘un‐instrumented’ canal at the terminus. Zuolo et al. (2015) demonstrated that Reciproc instruments achieved full working length in 85.63% of MB2 canals even without prior glide path preparation, compared to only 57.48% when hand files were used. These findings suggest that Reciproc facilitates achieving the full working length substantially and thereby improving patency, which is a critical factor affecting the outcome of the treatment. From the authors' perspective, achieving patency with hand and rotary instruments presented a significant challenge. Other studies have offered further support for this view by noting fewer procedural errors, faster treatments and less apical extrusion of debris with reciprocating instruments (Hamid et al., 2018; Jainaen et al., 2018; Nouroloyouni et al., 2023).

The large sample size in the current investigation made it possible to identify statistical significance even in subtle differences between predictors. For instance, it has been reported in literature that patients with existing periodontal problems were more likely to have a poor outcome of root canal treatments as a result of periodontal attachment loss (Caplan & Weintraub, 1997; Chang et al., 2024; Sarnadas et al., 2021). A recent study by López‐Valverde et al. (2023) found that the survival rate of RCT was lower in cases where the periodontal health of the affected tooth was in poor condition, particularly in cases with deep periodontal pockets. In other words, if a patient stays on top of their periodontal health by getting regular check‐ups and cleanings, the success rate of RCT will be higher. Our study findings align with this conclusion; it showed that patients who receive regular supportive periodontal treatments have a higher chance of retaining their teeth after root canal treatment. Regarding gender, some studies indicate that female patients are more concerned about their dental health than male patients. This difference may lead to fewer teeth being extracted after root canal treatments in female patients (Kim & Ahn, 2021; Thyvalikakath et al., 2022). Our study presented contrasting results, where there was no statistically significant difference between male and female patients. This finding agrees with another research performed to evaluate different factors affecting the outcome of RCT and implants (Doyle et al., 2007).

Several research studies have investigated the impact of patient age on the outcome of RCT. A prospective investigation by de Chevigny et al. (2008) concluded that there was no association between patient age and RCT. A similar result was also supported later by Thyvalikakath et al. (2022). Our study revealed that the probability of survival after RCT decreases with the increase in patient age. This indicates that the chances of teeth requiring extraction or retreatment after RCT rise by 2% for every year increase in patient age. This finding is supported by a retrospective study that was carried out on a huge number of cases (Kim & Ahn, 2021) and another study conducted by Chang et al. (2024). From our point of view, the lower incidence of failure in younger age might be attributed to the larger size of the pulp space, which allows easier instrumentation (Song et al., 2012). In addition to that, younger patients often have more robust immune systems, aiding in healing and reducing post‐operative inflammation (Weyand & Goronzy, 2016).

In this study, two more predictors were identified to significantly influence the outcome of the RCT. However, upon adjusting for potential biases using the EB method, these factors no longer had a discernible effect on the RCT outcome. The first of these factors was the number of previous RCTs the patient has undergone (in other teeth) before the tooth in question. Bartols et al. (2020) found that the need for a further intervention rises by 3% for each prior RCT a patient has undergone. Similarly, this follow‐up study indicates even a 5% increase in this risk per RCT. However, after adjusting for potential confounding factors using EB, this risk reduced to 0%. The second factor which showed a significant difference only in the unbalanced model was the experience of the treatment provider. In the present research study, 25% (3.523 teeth) of the total number of treated teeth were treated by an experienced dentist, while the remaining 75% (10.710 teeth) were treated by a group of non‐experienced practitioners. This ratio is close to the study of Burry et al. (2016) in which the percentage of teeth treated by a specialist was 31.5%. In the unbalanced model, we found that the outcome of the RCT was better when the treatment was provided by an experienced dentist, as the probability of untoward events was higher when the treatment was provided by general practitioners by 22%. The issue of the extent to which specialist therapy is advantageous remains a subject of debate, as the findings of our study are contradictory, in that the advantage of specialist therapy does not persist in the balanced model.

The current study also investigated the influence of several other predictors on the success of RCT, including tooth type, preoperative pulp status and whether the treatment has been completed in a single visit or multiple visits. However, the findings revealed that these factors do not play a statistically significant role in determining the treatment's outcome. For instance, the insignificant effect of tooth type aligns with a previous long‐term survival analysis (López‐Valverde et al., 2023). This contrasts with a systematic review, which showed that after RCT molars are more prone to fracture with subsequent extraction (Ng et al., 2010). These findings were supported by a cohort study mining the digital database of a German health insurance company with more than 550 000 root canal treatments (Raedel et al., 2015). Another large retrospective study showed that mandibular teeth, especially mandibular molars, are the most likely to be extracted after RCT (Kim & Ahn, 2021). Another factor that was found to play no significant role in the survival of teeth after RCT is the initial vitality status of the pulp. This finding is supported by the study of López‐Valverde et al. (2023) where they noticed in their retrospective analysis that the success of the treatment is linked to the absence of preoperative periapical pathosis rather than the vitality of the pulp. This is in contrast with studies conducted by Chugal et al. (2017) and Raedel et al. (2015). They found that the success was higher when the pulp of the affected tooth was vital. Finally, the difference in the outcome between single‐visit and multiple‐visit endodontic treatments was assessed. Our study showed that there is no statistically significant difference between the two protocols. This agrees with two systematic reviews that attempted to find differences between the two strategies (Manfredi et al., 2016; Schwendicke & Göstemeyer, 2017) in which their available data was inconclusive in determining if significant discrepancies between the two approaches are present. Nonetheless, this finding is important to a secondary finding in this study: While a much higher proportion of treatments in Protocol 3 could be treated in a single‐visit approach, the outcome was not affected.

The randomized controlled trial is widely recognized as the gold standard for conducting medical research to determine the efficacy of a treatment, facilitating its translation into clinical practice (Cuschieri, 2019). Despite their advantages, randomized controlled trials have some important limitations. For instance, they might not have enough statistical power to identify differences in rare effects between different treatment protocols. This is because they are often conducted with small groups of participants or over short periods, making it difficult to observe rare or long‐term side effects (Saldanha et al., 2022). They also often lack the statistical strength needed to detect subtle differences between different treatments or interventions, due to the limited sample sizes (Kostis & Dobrzynski, 2020). On the other hand, retrospective non‐interventional studies are rated at a lower level of evidence than randomized controlled trials based on the evidence‐based research systems (Durieux et al., 2013). With respect to that, a crucial step to minimize the bias in our study was to attempt to account for and balance confounders and covariates (Markoulidakis et al., 2023), which we tried to achieve by balancing the treatment protocols using two balancing methods, EB and GBLR, before performing the Cox proportional hazards analyses. EB is a data pre‐processing technique used in observational studies to ensure that treatment groups being compared have comparable characteristics. It involves adjusting the weights assigned to each observation, without removing any of them, in the data to match a set of predefined balance conditions (Hainmueller, 2012). It is a doubly robust method, which refers to its ability to provide unbiased treatment effect estimates even if the model used to balance covariates is mis‐specified or if the outcome model is mis‐specified for treatment groups (Zhao & Percival, 2017). The GBLR is a variation of the Generalized Boosted Models (GBM), which are effective machine learning algorithms used to estimate propensity scores for balancing treatment groups in observational studies which is achieved by iteratively fitting decision trees to optimize covariate balance (Griffin et al., 2023).

In our investigation, we also conducted a multistate analysis, which is a powerful statistical method used to characterize the progression of a set of individuals through a succession of intervention states until they come to a certain endpoint called the absorbing state (extraction of the tooth in this study). The difference between multistate and survival analyses is that in survival analysis, we evaluate the effect of a variable on the incidence of a single event. For this reason, it will not differentiate between the incidence of extraction or retreatment because they were all considered as one endpoint for the analysis. However, in multistate analysis we assess the effect of that specific variable on the incidence of each of the intervention states. Moreover, it will not stop at this point, because each tooth is followed up until the absorbing state (extraction), which helps to evaluate the effectiveness of surgical and non‐surgical retreatment (Le‐Rademacher et al., 2022; Matsena Zingoni et al., 2021; Meira‐Machado et al., 2009). Therefore, the method allows for the simultaneous analysis of multiple pathways of treatment interventions, providing a deeper understanding of the development and progression of diseases or the effects of different treatments (Le‐Rademacher et al., 2022). In line with the findings of a study by Bhagavatula et al. (2021), our study showed that extraction was the most commonly performed intervention after failure of non‐surgical root canal treatment, a finding which is applied also to the three treatment protocols individually, followed by non‐surgical retreatment and surgical retreatment. Additionally, both studies demonstrated that teeth that underwent surgical or non‐surgical retreatment had an increased risk of being extracted than teeth that did not. This highlights the potential risks associated with retreatment and underscores the importance of initial treatment success. An explanation might be that the prognosis of these teeth was already questionable and RCT was conducted as a last resort to save the tooth, but it eventually failed. Another common interesting finding was that the transition from non‐surgical retreatment to surgical retreatment mostly occurred within the first two years after the retreatment and it was hardly observed after that. Regarding the effect of the treatment protocol on the time to the next transition, our current investigation shows that the probability of extraction and retreatment (both surgical and non‐surgical) is lower when Protocol 3 was used (Figure 9). However, no statistically significant differences were observed between Protocol 1 and Protocol 3 across other transitions. On the other hand, Protocol 2 had a significantly lower hazard ratio in the transition from initial treatment to non‐surgical retreatment when compared to Protocol 1, while in other transitions the difference was not significant. It is also worth mentioning that the transition from initial treatment to extraction in Protocol 2 had a slightly, but not significant, higher hazard ratio when compared to Protocol 1.

The typical limitations of retrospective studies can be applied to our study (Talari & Goyal, 2020). For instance, retrospective studies rely on patient charts or records that were not designed to be included in medical research, which might contain missing data. Bias also may arise from non‐randomized patient selection, uneven recall and lost patient follow‐up, which may lead to information bias (Ramirez‐Santana, 2018). The bias that resulted from the lack of randomization was overcome to a great extent by the application of the covariate balancing methods we utilized, while we tried to minimize the effect of the information bias as much as possible by including all available baseline characteristics as covariates, which were later balanced. However, some covariates and baseline characteristics could not be balanced for technical reasons. For example, it was not possible for this study to obtain information about whether or not the treated teeth received a full coverage crown after finishing the treatment, while many scientific studies concluded that the application of crown coverage increases the survival rates of the treated tooth (Aquilino & Caplan, 2002; Chang et al., 2024; Ng et al., 2010; Thyvalikakath et al., 2022). Additionally, it was not possible to differentiate between primary or secondary RCT as the initial treatment. However, studies have shown there is no difference in the outcome between both types of treatments (Mareschi et al., 2020; Stoll et al., 2005). Moreover, the database on which this study was conducted did not provide information about the general health condition of the treated patients, while several health conditions have been found to affect the outcome of RCT. For instance, Laukkanen et al. (2019) came to a conclusion that patients being treated for diabetes mellitus were more liable for extraction of their endodontically treated teeth, a finding which was agreed with in a systematic review (Segura‐Egea et al., 2023). Moreover, it was not possible to radiographically assess the periapical condition of the treated teeth or the quality of root canal filling, which could have had an effect on the outcomes (Ng, Mann, & Gulabivala, 2008; Ng, Mann, Rahbaran, et al., 2008). The type of restoration also could not be added to the analysis because this information was not available in the database. Additionally, the reason behind further intervention was not recorded and hence was not added to the analysis. Finally, certain variables, such as use of apex locators, Chlorhexidine and EDTA, could not be added to the analysis because there was no reference to these procedures in the database query.

Despite there being some limitations, the study has some advantages that help to make its findings stronger and more reliable. The inclusion of a large number of teeth and the long period of observation in the study adds to the statistical power and generalizability of the discoveries. Moreover, using the balancing methods decreases bias and effects of confounding variables and improves the comparability of the treatment protocols. In addition, the clear timelines and procedural steps of each protocol allow for their separation and hence accurate analysis despite the retrospective nature of the study. Also, the application of sensitivity tests adds another level of validity to the findings.

## CONCLUSION

Within the limitations of this study, a comprehensive treatment protocol (Protocol 3) including the use of an apex locator, the Reciproc system, ultrasonic irrigation and EDTA demonstrated superior outcomes in improving tooth retention and tooth retention without further interventions like extraction, retreatment, or surgical retreatment after RCT. This protocol decreased the need for further interventions by approximately 30% to 40% when compared to the other protocols. Furthermore, we conclude that patient age and oral hygiene maintenance measures are important predictors of tooth survival following RCT. Further research is encouraged to explore the exact mechanisms underlying the superior performance of treatment Protocol 3.

## AUTHOR CONTRIBUTIONS

*Conceptualization*: Andreas Bartols. *Data curation*: Andreas Bartols and Ahmed Elmaasarawi. *Formal analysis*: Ahmed Elmaasarawi and Andreas Bartols. *Investigation*: Andreas Bartols and Ahmed Elmaasarawi. *Methodology*: Andreas Bartols and Ahmed Elmaasarawi. *Project administration*: Andreas Bartols and Mohamed Mekhemar. *Resources*: Andreas Bartols and Mohamed Mekhemar. *Software*: Ahmed Elmaasarawi. *Supervision*: Andreas Bartols and Mohamed Mekhemar. *Validation*: Andreas Bartols and Ahmed Elmaasarawi. *Visualization*: Ahmed Elmaasarawi. *Writing—original draft*: Ahmed Elmaasarawi. *Writing—review & editing*: Ahmed Elmaasarawi, Mohamed Mekhemar and Andreas Bartols.

## FUNDING INFORMATION

This retrospective cohort study was undertaken with no external funding. The authors self‐funded the whole study project, including data collecting, analysis, interpretation and publication writing.

## CONFLICT OF INTEREST STATEMENT

The authors declare no conflicts of interest related to this study.

## ETHICAL STATEMENT

Our determined dedication to maintaining the highest ethical standards in research is affirmed in our submission to the International Endodontic Journal. Internationally recognized principles, such as the Declaration of Helsinki and the Belmont Report, were strictly adhered to during the conduct of our study. We ensured adherence to data protection standards and secured all required ethical approvals from the pertinent institutional review boards. Moreover, the study protocol received ethical approval from the Institutional Review Board of the University of Kiel (IRB No. D 495/24).

## PATIENT CONSENTS

Considering this study comprised a retrospective examination of previously anonymized data, patient consent was not required for participation. All data were collected and processed per applicable privacy and ethical standards to ensure confidentiality and regulatory compliance.

## REPORTING GUIDELINES

This investigation was written following the guidelines, instructions and recommendations of ‘STrengthening the Reporting of Observational Studies (STROBE)’ and ‘Preferred Reporting items for OBservational studies in Endodontics (PROBE)’.

## Data Availability

The data from this retrospective cohort study are available upon reasonable request. Due to privacy and ethical concerns, the dataset cannot be made public. Researchers interested in accessing the data for replication, validation, or further analysis should contact the corresponding author with questions about data availability and access. The institutional review board will assess requests to ensure they comply with data protection legislation and ethical principles.
